# Cardiac metabolism as a driver and therapeutic target of myocardial infarction

**DOI:** 10.1111/jcmm.15180

**Published:** 2020-05-08

**Authors:** Coert J. Zuurbier, Luc Bertrand, Christoph R. Beauloye, Ioanna Andreadou, Marisol Ruiz‐Meana, Nichlas R. Jespersen, Duvaraka Kula‐Alwar, Hiran A. Prag, Hans Eric Botker, Maija Dambrova, Christophe Montessuit, Tuuli Kaambre, Edgars Liepinsh, Paul S. Brookes, Thomas Krieg

**Affiliations:** ^1^ Department of Anesthesiology Laboratory of Experimental Intensive Care and Anesthesiology Amsterdam Infection & Immunity Amsterdam Cardiovascular Sciences Amsterdam UMC University of Amsterdam Amsterdam The Netherlands; ^2^ Institut de Recherche Expérimentale et Clinique Pole of Cardiovascular Research Université catholique de Louvain Brussels Belgium; ^3^ Cliniques Universitaires Saint‐Luc Brussels Belgium; ^4^ Laboratory of Pharmacology Faculty of Pharmacy National and Kapodistrian University of Athens Athens Greece; ^5^ Department of Cardiology Hospital Universitari Vall d’Hebron Vall d’Hebron Institut de Recerca (VHIR) CIBER‐CV Universitat Autonoma de Barcelona and Centro de Investigación Biomédica en Red‐CV Madrid Spain; ^6^ Department of Cardiology Aarhus University Hospital Aarhus N Denmark; ^7^ Department of Medicine University of Cambridge Cambridge UK; ^8^ Pharmaceutical Pharmacology Latvian Institute of Organic Synthesis Riga Latvia; ^9^ Department of Pathology and Immunology University of Geneva School of Medicine Geneva Switzerland; ^10^ Laboratory of Chemical Biology National Institute of Chemical Physics and Biophysics Tallinn Estonia; ^11^ Department of Anesthesiology University of Rochester Medical Center Rochester NY USA

**Keywords:** ischemia, metabolism, mitochondria

## Abstract

Reducing infarct size during a cardiac ischaemic‐reperfusion episode is still of paramount importance, because the extension of myocardial necrosis is an important risk factor for developing heart failure. Cardiac ischaemia‐reperfusion injury (IRI) is in principle a metabolic pathology as it is caused by abruptly halted metabolism during the ischaemic episode and exacerbated by sudden restart of specific metabolic pathways at reperfusion. It should therefore not come as a surprise that therapy directed at metabolic pathways can modulate IRI. Here, we summarize the current knowledge of important metabolic pathways as therapeutic targets to combat cardiac IRI. Activating metabolic pathways such as glycolysis (eg AMPK activators), glucose oxidation (activating pyruvate dehydrogenase complex), ketone oxidation (increasing ketone plasma levels), hexosamine biosynthesis pathway (O‐GlcNAcylation; administration of glucosamine/glutamine) and deacetylation (activating sirtuins 1 or 3; administration of NAD^+^‐boosting compounds) all seem to hold promise to reduce acute IRI. In contrast, some metabolic pathways may offer protection through diminished activity. These pathways comprise the malate‐aspartate shuttle (in need of novel specific reversible inhibitors), mitochondrial oxygen consumption, fatty acid oxidation (CD36 inhibitors, malonyl‐CoA decarboxylase inhibitors) and mitochondrial succinate metabolism (malonate). Additionally, protecting the cristae structure of the mitochondria during IR, by maintaining the association of hexokinase II or creatine kinase with mitochondria, or inhibiting destabilization of F_O_F_1_‐ATPase dimers, prevents mitochondrial damage and thereby reduces cardiac IRI. Currently, the most promising and druggable metabolic therapy against cardiac IRI seems to be the singular or combined targeting of glycolysis, O‐GlcNAcylation and metabolism of ketones, fatty acids and succinate.

## 1 INTRODUCTION

Cardiac metabolism changes rapidly during a sudden ischaemic episode of the heart, with the oxygen shortage repressing oxidative metabolism of fatty acids (FA), carbohydrates, ketones and amino acids, and activating anaerobic glycolysis to spare the use of limited oxygen. During reperfusion with the wash‐in of oxygen and the wash‐out of ischaemic metabolites, there is an abrupt normalization of intracellular pH and a specific start‐up of oxidative metabolism for the various substrates, with ongoing changes in what is now aerobic glycolysis. These metabolic changes during ischaemia and early reperfusion are not merely passive bystanders, but determine to a large extent the actual injury developing in the heart following an ischaemic episode. Modulation of these metabolic changes, therefore, offers the opportunity for developing therapy against cardiac IRI, as was already shown by Sodi‐Pallares in 1962 using potassium‐insulin‐glucose administration during myocardial infarction.^1^ Since these earlier studies, it has become clear that metabolic therapy for IRI has travelled a rather bumpy road, without the development of a proven effective clinical metabolic therapy as of yet. Here, we review the current literature concerning this topic, going from the well‐known metabolic pathways to novel metabolic targets that go beyond the general metabolism of glucose and FA, and focus on important processes such as acidosis, ketone oxidation, succinate accumulation, mitochondrial F_O_F_1_‐ATPase, energy transfer pathways, protein O‐GlcNAcylation and acetylation as novel metabolic targets for treating IRI.

## 2 GENERAL ASPECTS OF CARDIAC METABOLISM IN HEALTHY HEART

**Figure 1:**
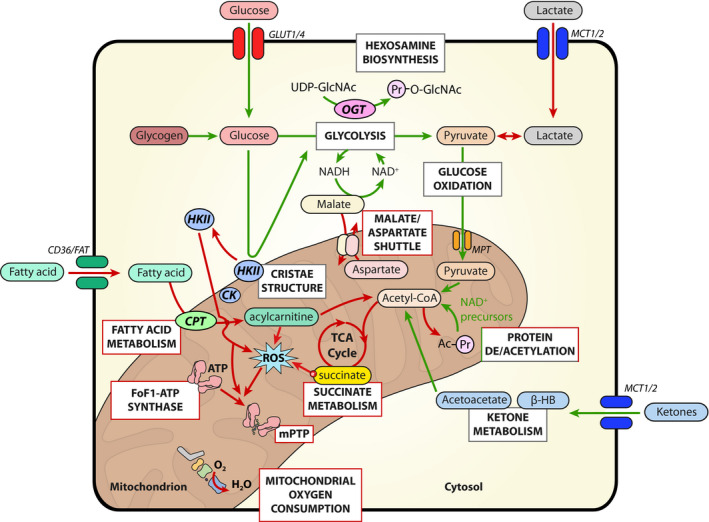
Summary of the proposed pathways of cardiac metabolism covered in this review. Many of the discussed pathways show protection against IRI (green arrows) or are protective if blocked (red arrows). CK, creatine kinase; CPT, carnitine palmitoyltransferase; HKII, hexokinase II; MCT, monocarboxylate transporter; MPT, mitochondrial pyruvate transporter; mPTP, mitochondrial permeability transition pore; OGT, O‐GlcNAc transferase

The healthy heart is a true omnivore in that it can degrade various energy‐containing substrates. The major cardiac fuels for respiration are fats (triglyceride and long‐chain fatty acids), carbohydrates (glucose, lactate and cardiac glycogen) and ketone bodies (acetoacetate and β‐hydroxybutyrate) (Figure 1). Regulation of substrate use by the heart is to a large extent determined by the amount of substrate delivered to the heart (ie plasma concentration), the number of specific substrate transporters present in the cell membrane (CD36/Fat for fatty acids, GLUT1/4 for glucose and MCT1/2 for lactate and ketone bodies) and the activities of the metabolic enzymes and substrate/products/cofactors present in the enzymatic pathways.^2^, ^3^, ^4^ It should thereby be realized that a high plasma concentration of one substrate usually competes and inhibits the use of other substrates. For example, high plasma fatty acid levels will impair glycolysis and glucose oxidation,^4^ or increasing plasma lactate levels will impair fatty acid oxidation and glycolysis.^5^ Although in general fatty acids contribute more than carbohydrates to ATP generation in the heart, this depends critically on (patho) physiological, nutritional and hormonal state. For example, when insulin, lactate and fatty acids are present at normal physiological concentrations in the ex vivo‐perfused rodent heart, the contribution of carbohydrates can be higher than that of fatty acids.^6^, ^7^, ^8^ Ketone bodies, when provided at physiological plasma concentrations (<0.3 mmol/L), contribute less than 5% to cardiac ATP generation.^9^ Each substrate is finally broken down to acetyl coenzyme A (acetyl‐CoA) that feeds the tricarboxylic acid (TCA) cycle to produce reducing equivalents (NADH and FADH_2_) that then feed the electron transport chain to build a proton gradient to drive the F_O_F_1_‐ATP/synthase to make ATP. During normoxia, approximately 90% of the ATP produced is derived from mitochondrial oxidative breakdown of substrates, with cytosolic glycolysis only contributing ~5%‐10% of total ATP production. Only during total ischaemia does glycolysis become the major supplier of ATP, when glycogen stored in the heart starts feeding glycolysis. The malate/aspartate shuttle (MAS; Figure 1) is critical for maintaining glycolytic rate because it is the main pathway to recycle glycolysis‐produced NADH through the mitochondria into cytosolic NAD^+^ to maintain glycolytic activity.

It should be noted that for cardiac metabolism, it is not only the flux through the metabolic pathway that regulates cardiac function and physiology**,** but also the level of its intermediary metabolites. Typical examples of regulating metabolic intermediates are as follows: a) acetyl‐CoA, partly regulating the acetylation status and thereby function of proteins, b) succinate, build‐up during ischaemia, partly determining reactive oxygen production upon reperfusion, and c) UDP‐GlcNAc, as end product of the hexosamine biosynthetic pathway. Although only a small part of glucose (<0.01% of glycolytic rate)^10^ is shuttled into this pathway, the generated O‐linked attachment of N‐acetylglucosamine moiety onto proteins affects protein function significantly.

Finally, spatial and temporal distribution of metabolic enzymes can also be important effectors of cardiac metabolism. One classical example relates to hexokinase II (HKII) that shuttles between mitochondria and cytosol, depending on nutritional and pathophysiological status. When bound to mitochondria (mtHKII), HKII increases glycolysis, impairs in vivo cardiac oxygen consumption, impairs fatty acid oxidation, facilitates growth processes and protects mitochondria against injury.^7^, ^11^, ^12^ Another example is creatine kinase (CK), the enzyme making ATP from phosphocreatine (PCr) and thereby functioning as an important local buffer for ATP breakdown. Cytosolic and mitochondrial isoforms of CK form a metabolic signalling network that is needed for rapid energy (ATP) transfer between ATP producers (mitochondria) and users (muscle contraction and ion pumps), preventing measurable decreases in ATP during changes in cardiac work.^13^, ^14^

## 3 TARGETING METABOLIC PATHWAYS TO COMBAT CARDIAC IRI

### 3.1 Glycolysis

#### 3.1.1 Glycolysis during ischaemia

During ischaemia, glycolysis is increased due to (i) increased glucose extraction from the blood (going from 1% during normoxia to 30% extraction during severe low flow), (ii) increased translocation of GLUT transporters to the sarcolemma, (iii) increased glycogenolysis, due to stimulation of glycogen phosphorylase A by diminishing glucose, ATP, glucose 6‐phosphate and increases in AMP and Ca^2+^, (iv) removing citrate‐mediated inhibition of the glycolysis‐controlling enzyme 6‐phosphofructokinase‐1 (PFK‐1) and (v) AMP‐activated protein kinase (AMPK) activation, resulting in both increasing GLUT transport capacity at the membrane and activation of 6‐phosphofructokinase‐2 (PFK2), resulting in fructose 2,6‐bisphosphate production, the most potent allosteric stimulator of PFK1.^15^, ^16^, ^17^, ^18^ Activation of glycolysis during low‐flow conditions makes use of all these mechanisms, whereas, during no‐flow conditions, only mechanisms (iii), (iv) and (v) are active. Of note, although most pre‐clinical studies have employed the no‐flow ischaemia model, low‐flow ischaemia may better reflect the clinical condition of myocardial infarction, where residual flow is often present.^19^ Following long‐lasting glycolytic activity, glycolysis can subsequently be inhibited by accumulation of its products: lactate, protons and NADH.^15^ As long as glycolysis can proceed and generate ATP during the entire oxygen‐limited condition, it offers protection against IRI. Because during low‐flow conditions lactate and protons can still partly be removed and glucose uptake proceeds, inhibition of glycolysis is much delayed as compared to no‐flow conditions. This explains why during even extreme low‐flow ischaemia (5% of normal flow), an extended 40‐min period of low‐flow induces hardly any IRI (<3% cell death),^20^ whereas 40‐minute no‐flow induces 20x more cell death.^21^ Low‐flow ischaemia does induce damage when glucose is replaced by another substrate or taken out of the perfusate, supporting the concept that it is the glycolytically derived ATP during ischaemia that protects against IRI.^22^, ^23^ Increasing glycolysis during low‐flow ischaemia through, for example, increasing levels of glucose and insulin in the perfusate or boosting endogenous glycogen is associated with delayed contracture development and protection against IRI.^24^ The start of contracture development during ischaemia is a read‐out of the time when glycolysis stops^22^ and the free energy of ATP hydrolysis, the ∆*G*_ATP_, falls below a critical level to support the ATPase activity of ion pumps and cross‐bridge cycling.^26^ Also during no‐flow ischaemia activating or prolonging glycolysis is most frequently associated with protection against IRI. In line, AMPK‐deficient mouse models are characterized by decreased glucose uptake and glycolysis, increased ATP depletion during ischaemia, more rapid and severe ischaemic contracture, increased cell death during reperfusion and poorer post‐ischaemic contractile recovery.^27^, ^28^ Previous studies also clearly showed that either increasing pre‐ischaemic glycogen^29^ or activating glycolysis through redox control by niacin‐induced lowering of NADH/NAD^30^ resulted in delayed ischaemic contracture and reduced IRI. Similarly, the pharmacologic overactivation of AMPK reduces cardiomyocyte death and ameliorates post‐ischaemic function recovery.^31^, ^32^

However, there are experimental conditions where increasing pre‐ischaemic glycogen and/or a delayed contracture development were actually associated with increased IRI.^23^, ^33^, ^34^ Although it is not completely clear what sets these conditions apart, it could be related to excessive glycogen loading (and thus excessive accumulation of glycogen breakdown products and consequently low pH during ischaemia)^23^ due to elevated insulin before ischaemia (thereby inhibiting non‐ischaemic glycogen breakdown)^16^ as compared to no insulin before ischaemia.^33^ The presence of more insulin before ischaemia can also result in impaired activation of the reperfusion injury salvage kinase (RISK) pathway during reperfusion,^35^ possibly explaining the increased IRI in these experimental conditions. Mechanisms explaining increased infarct size with increased accumulation of glycolytic breakdown products during ischaemia are a) detachment of hexokinase II from mitochondria (mtHKII) due to high levels of G6P and low pH^11^, ^33^, ^36^ and/or b) increased ischaemic Na^+^ loading due to increased proton production by excessive glycogen breakdown, resulting in increased Ca^2+^ overload upon reperfusion.^23^

#### 3.1.2 Glycolysis during reperfusion

The strongest cardioprotective intervention for protecting against cardiac IRI, observed across all species, concerns ischaemic pre‐conditioning (IPC). IPC relates to the application of short, non‐lethal periods of ischaemia before the long, lethal period of ischaemia, resulting in a 75% reduction in infarct size.^37^ IPC activates glycolysis during normoxia and reperfusion, through activation of AMPK and Akt, translocation of GLUT4 transporters to the cell membrane and translocation of HKII from cytosol to mitochondria (Figure 1).^7^, ^38^, ^39^, ^40^, ^41^, ^42^, ^43^ Interestingly, no protection by IPC is observed in the absence of glucose.^39^ Taken together, the data strongly suggest that IPC protective mechanism is mediated through activated aerobic glycolysis during early reperfusion. An increased glycolysis may mediate protection through increased activity of mtHKII, knowing that glucose phosphorylation by mtHKII is needed for protection against mitochondrial damage and cell death.^44^, ^45^ Additionally, older literature has suggested that protection by glycolysis is due to the preferential use of glycolytically produced ATP by ion pumps and restoration of ionic homeostasis during reperfusion.^46^ Finally, increasing glycolysis during early reperfusion contributes to maintain a low pH, which in turn exerts multiple protective effects, including the prevention of mitochondrial permeability transition pore (mPTP) opening,^47^ one of the mechanisms proposed for the cardioprotective effect of post‐conditioning.^48^ It is especially an increased ratio of glycolysis over glucose oxidation that induces this net proton production,^49^ and may, for example, explain the decreased infarct size following IR in high‐fat diet‐induced obesity.^50^ It is thereby noteworthy that this reduction in infarct size with increased glycolysis uncoupling from glucose oxidation is opposite to the negative effect this uncoupling exerts on cardiac mechanical function, resulting in a decreased cardiac performance and mechanical efficiency.^49^ However, the decreased mechanical function upon reperfusion may actually contribute to the reduction in infarction, knowing that a slow recovery of energy requirement in early reperfusion is cardioprotective^51^ and that there is often a dichotomy between recovery of mechanical function and cell death following IR.^52^, ^53^

Besides ‘conditioning interventions’ to activate aerobic glycolysis, several pharmacologic cardioprotective agents are also known to increase glucose uptake/glycolysis (metformin, insulin, volatile anaesthetics, adenosine, NO donors, fructose 1,6‐diphosphate, nicotinamide mononucleotide (NMN), HIF1α stabilizers and AMPK activators). Some of these agents should ideally be applied at the end of the ischaemic period or at the onset of reperfusion, in order to prevent excessive glycogen accumulation, resulting in excessive accumulation of glycogen breakdown products during the ischaemic episode, thereby offsetting the beneficial effects of an activated glycolysis during early reperfusion.

In summary, most studies report that increasing glycolysis during ischaemia and early reperfusion confers protection against cardiac IRI, making glycolysis a potential important metabolic goal for IRI therapy.

### 3.2 Hexosamine biosynthesis pathway (HBP)

Beyond glycolysis, glucose can be used by accessory metabolic pathways such as the hexosamine biosynthesis pathway (HBP) (Figure 1). HBP concludes with O‐linked attachment of N‐acetylglucosamine moiety (O‐GlcNAc) onto serine and threonine residues of proteins. Similar to other post‐translational modifications, O‐GlcNAcylation is a dynamic and reversible molecular process involved in the regulation of metabolism, gene expression and protein‐protein interaction.^54^, ^55^ The rate of O‐GlcNAcylation is governed by (extra)cellular stresses or stimuli but also by nutrient type and availability. Next to glucose, glutamine, but also many other molecules, such as acetyl‐CoA and exogenous glucosamine, can enter into HBP to promote O‐GlcNAcylation.^56^, ^57^

Increases in O‐GlcNAcylation level occur and appear deleterious under chronic situations such as diabetes and cardiac hypertrophy.^54^, ^55^ On the other hand, it has been shown that O‐GlcNAcylation is central for the maintenance of cardiovascular functions. Cardiac‐specific deletion of the gene encoding for the O‐GlcNAc transferase (OGT), the enzyme responsible for the addition of O‐GlcNAc moiety on proteins, leads to progressive cardiomyopathy.^58^ More importantly, increases in protein O‐GlcNAcylation are overall protective under acute settings. Using various in vitro, ex vivo and in vivo animal models, numerous studies demonstrate that O‐GlcNAcylation confers cardioprotection following acute IRI and other types of cardiac injuries.^54^, ^59^ Among the in vitro studies, protocols that mimic IR in isolated cardiomyocytes have been shown to promote protein O‐GlcNAcylation, and further increase in O‐GlcNAcylation using glucosamine treatment or OGT overexpression decreases cell death.^60^, ^61^ In line, IR performed in isolated perfused hearts increases O‐GlcNAc levels and, once again, treatment promoting O‐GlcNAcylation (glucosamine but also inhibitors of the β‐N‐acetylglucosaminidase OGA, the enzyme responsible for removing O‐GlcNAc moiety) reduces ischaemic contracture and improves post‐ischaemic contractile function recovery.^62^, ^63^, ^64^, ^65^ Notably, the treatment was also cardioprotective when applied at the onset of reperfusion, which increases its clinical relevance.^66^ Finally, IPC performed in the ex vivo‐perfused heart or in vivo by left anterior descending artery ligation stimulates protein O‐GlcNAcylation and administration of an OGA inhibitor prior to surgery reduces infarct size.^66^, ^67^ This was further validated in humans submitted to remote IPC.^68^ These data suggest that the cardioprotective effect of IPC may be mediated at least in part by an increase in OGT expression and activity.^67^

The molecular mechanisms involved in the cardioprotective action of O‐GlcNAcylation are not fully understood. It has several effects, including (i) the modification of Ca^2+^ handling,^64^ (ii) the increase in the mitochondrial translocation of the anti‐apoptotic Bcl‐2 protein,^69^ (iii) the alteration of P38 MAPK signalling^63^ and (iv) the attenuation of mitochondrial depolarization and mPTP opening, among others. Importantly, the cardioprotective action of O‐GlcNAcylation is lost under diabetic conditions, showing that the action of this post‐translational modification could differentially affect cardiac function when acutely or chronically induced.^54^

In summary, most studies report that the acute increase in O‐GlcNAcylation elicits protection against cardiac IRI. The cardioprotective action of O‐GlcNAcylation could be promoted by acting pharmacologically on O‐GlcNAc enzymes such as OGA or OGT or by metabolically fuelling HBP via glucosamine and/or glutamine. It would be worthwhile to investigate these novel strategies in humans.

### 3.3 The malate‐aspartate shuttle

In the healthy adult myocardium, the malate‐aspartate shuttle (MAS) constitutes the main pathway for transportation of redox products from glycolysis in the cytosol into the mitochondrial matrix over the impermeable inner mitochondrial membrane (Figure 1).^70^ Under normal conditions, the shuttle capacity is high. The shuttle also meets elevated glycolytic demand in a variety of physiological and pathophysiological processes including cardiac hypertrophy.^71^ Hence, shuttle activity is modifiable. Because of its central role as a regulatory mechanism in the energy metabolism of the cardiomyocytes, it constitutes a potential target for induction of cardioprotection.

The MAS has gained conceptual interest as a means to modify mitochondrial function, because transient shut down of metabolism by blocking the cytosolic‐mitochondrial crosstalk via the MAS may induce cardioprotection (Figure 1). As a proof of concept, transient aminooxyacetate (AOA) administration can induce reversible MAS inhibition. AOA is a non‐specific competitive inhibitor of various amino acid transaminases^72^ but in in situ heart models, AOA primarily leads to reversible inhibition of the MAS.^73^, ^74^ However, recent data also show that AOA directly reacts with alpha‐keto acids (pyruvate, alpha‐ketoglutarate, oxaloacetate, etc) to form stable oximes with unknown functional consequences as of yet.^75^

Cardioprotection by MAS inhibition using AOA can be induced both prior to and during an ischaemic insult.^76^, ^77^ In the normal heart, MAS inhibition reduces mitochondrial complex I‐linked respiration and glycolysis.^76^ Even though the effect of MAS inhibition predominantly involves the glycolytic pathway, MAS inhibition also yields cardioprotection in a setting with free FA as additional substrate next to glucose.^78^ MAS inhibition reduced succinate‐induced ROS production (see 3.5.5. for mechanism) by limiting transport of substrate to the succinate build‐up.^79^ The attenuated succinate oxidation during reperfusion reduces reverse electron flow at complex I and hence reduces oxidative stress and cellular damage. This key mechanism is further supported by the ability of MAS inhibition to preserve post‐ischaemic complex I respiration.^77^

However, the mechanism does not explain that MAS inhibition also reduces infarct size when administrated in the late phase of an ischaemic event, when it is unlikely to reduce succinate substantially.^77^ MAS inhibition may activate other mechanisms, depending on the timing of administration. The main effect of AOA is inhibition of aspartate aminotransferase, which is the pivotal enzyme in the MAS. The aspartate aminotransferase uses oxaloacetate as substrate, and inhibition by AOA may increase the intramitochondrial concentration of this tricarboxylic acid cycle intermediate. Because oxaloacetate serves as a strong inhibitor of succinate dehydrogenase thereby limiting post‐ischaemic oxidation of succinate, it is of interest to study whether the endogenously formed oxaloacetate conveys protection through reduced succinate dehydrogenase activity during reperfusion.

The MAS constitutes an important regulatory mechanism in the cardiomyocyte. The MAS may serve as a target for cardioprotection. However, drug toxicity remains a challenge and development of new reversible MAS inhibitors is necessary.

### 3.4 Lysine acetylation of cardiac proteins

The adduction of acyl groups to protein lysine residues (acylation) is a biologically significant post‐translational modification. Although many such acylations exist (eg succinylation, glutarylation and malonylation), here we focus on the most widely studied, acetylation. Early studies on the cardiac acetyl‐proteome revealed a preponderance of metabolic and mitochondrial targets,^80^, ^81^ leading to speculation that lysine acetylation is an important metabolic regulator. However, recent studies in hyperacetylation models have revealed a minimal impact on bioenergetics,^82^ consistent with studies at the molecular level.^83^ An important consideration in this regard is acetylation stoichiometry, with little understood about the precise relationship between site occupancy and enzyme activity.

While there are thousands of kinases and phosphatases in a typical cell, enzymes that regulate acetylation number far fewer. Most work on acetyltransferases has focused on those in the nucleus that regulate histones (see Ref. ^84^ for recent review). However, more recently other acetylation pathways have been uncovered. In particular, GCN5L1 was identified as a mitochondrial lysine acetyltransferase expressed in the heart,^85^ and its deletion confers a number of cardiac pathologic phenotypes.^86^ Furthermore, direct non‐enzymatic lysine acetylation from acetyl‐CoA (and corresponding acylation from other acyl‐CoAs) has been found to occur in the cell,^87^ such that the concept of ‘acyl‐carbon stress’ is now well established to be partly mediated by excessive lysine acetylation.^88^, ^89^

On the deacetylation side, in addition to the histone deacetylases (HDACs), the sirtuin (SIRT) class of NAD^+^‐dependent lysine deacylases has emerged as key mediators of cardioprotection (Figure 1). Of the 7 mammalian SIRTs, SIRT1 and SIRT3 are robustly expressed in the heart and have been shown to play a direct role in several cardioprotective paradigms.^39^, ^90^, ^91^, ^92^, ^93^, ^94^ Unlike other tissues where SIRT1 is mostly nuclear, cardiomyocyte SIRT1 resides mostly in the cytosol^91^ and is thus positioned to interact with the cardioprotective signalling machinery.

Inhibition of SIRT1 either pharmacologically^91^ or genetically^39^, ^90^ prevents cardioprotection via ischaemic pre‐conditioning (IPC). Conversely, activation of SIRT1 either pharmacologically^95^, ^96^, ^97^ or genetically^39^ is sufficient to confer cardioprotection against acute IRI. Furthermore, a decline in SIRT1 activity is proposed to play a role in the loss of IPC protection with ageing.^98^ In the context of metabolism, it was shown that the metabolic remodelling that occurs in acute IPC is critically dependent on SIRT1.^99^ SIRT1 is also proposed to be an intermediate signal in cardioprotection via other stimuli such as phosphodiesterase (PDE) inhibitors.^10^ Given the original discovery of the sirtuins as putative mediators of the longevity effects of caloric restriction (CR)^11^ it has also been posited that the cardioprotective benefits of CR may be mediated via SIRT1.^12^ Similar cardioprotective benefits have also been described for mitochondrial SIRT3,^92^, ^93^ with its pharmacologic activation also shown to confer acute cardioprotection.^13^ Among the more prominent SIRT3 deacetylation targets is the mPTP regulator cyclophilin D (CypD), with deacetylation at lysine 166 (mouse) required for the cardioprotective effects of SIRT3.^14^

Although activation of SIRT1 or SIRT3 may appear attractive as a pharmacologic protective strategy, it should be cautioned that the specificity of many SIRT‐activating drugs is uncertain, with stilbenes such as resveratrol plus several commercial SIRT1‐activating drugs called into question.^15^ In addition, inactivation of the NAD^+^‐recycling enzyme NAMPT phenocopies SIRT1 inhibition in preventing cardioprotection,^93^, ^106^ suggesting that NAD^+^ availability is a critical determinant for the cardioprotective efficacy of SIRT1. However, while the potential of boosting NAD^+^ bioavailability via delivery of precursors such as nicotinamide mononucleotide (NMN) or nicotinamide riboside (NR) has shown considerable promise in pre‐clinical studies,^95^, ^107^ the wide variety of non‐SIRT metabolic pathways that rely on the NAD^+^/NADH redox couple^18^ suggests that side effects of such compounds may outweigh their cardioprotective benefits.^19^ Interestingly, it may well be that part of the cardioprotective effects of NAD^+^‐boosting strategies mainly involves redox‐controlled activation of glycolysis.^19^ In line, we recently showed that increased protein acetylation by metabolic over‐fuelling dramatically reduced both insulin‐ and AMPK‐mediated glucose uptake in cardiomyocytes, presuming the importance of reducing protein acetylation for promoting glucose metabolism during IRI.^110^, ^111^ Overall, while promoting deacetylation, for example by targeting SIRT1 activation, is an attractive avenue for cardioprotection, considerable effort is required for the development of specific drugs to achieve this end.

### 3.5 Mitochondrial metabolism

#### 3.5.1 Oxygen consumption

A general characteristic of irreversible ischaemia, that is long (≥25 minutes) periods of ischaemia with cell death occurring, is the fast and complete recovery of oxygen consumption and therefore metabolic recovery during early reperfusion. At the same time, the recovery of mechanical function is lagging behind and still severely depressed. Thus, there is metabolic recovery that is uncoupled from mechanical recovery. In contrast, with reversible ischaemia, thus short (≤20 minutes) periods of ischaemia without cell death, recovery of oxygen consumption is much slower and still coupled to mechanical function following reversible ischaemia.^112^, ^113^, ^114^ This fast recovery of post‐ischaemic metabolism was associated with increased mitochondrial Ca^2+^ in early reperfusion with irreversible ischaemia.^112^, ^113^, ^114^, ^116^ The fast recovery of oxygen consumption can possibly be explained by Ca^2+^ activation of pyruvate dehydrogenase, resulting in increased carbohydrate (glucose, lactate, pyruvate) oxidation during the first hour of reperfusion.^116^ At high FA levels (>1.0 mmol/L), increased glucose oxidation is not observed,^117^ likely because high FA impair pyruvate dehydrogenase (PDH) activation. Thus, fast metabolic recovery uncoupled from mechanical function is a signature of damaging irreversible IR, although it is unknown whether this is causal to, or just an epiphenomenon of, IRI. When causal, it offers the possibility that imposing slower metabolic recovery in early reperfusion will protect from IRI. Indeed, interventions directed at an attenuated recovery of blood flow,^118^ or impaired mitochondrial activity by rotenone or mitochondrial complex I by nitrosating/NO agents are protective,^120^, ^121^, ^122^ supporting the notion of slow metabolic wake‐up^51^ as a primary mechanism to combat IRI.

#### 3.5.2 Glucose oxidation

Glucose oxidation, which actually is oxidation of the end product of glycolysis, that is pyruvate, occurs in the mitochondria. The flux from glycolysis to glucose oxidation is mostly controlled by the activity of the PDH complex located in the mitochondrial matrix. Glucose oxidation is completely shut down during ischaemia; its degree of reactivation during reperfusion depends, for example, on the availability of FA for oxidation because FA compete with glucose for oxidation. During early reperfusion, FA oxidation is increased due to activation of AMPK by the ischaemia.^123^, ^124^ FA oxidation reduces glucose oxidation because of the Randle cycle, in which acetyl‐CoA derived from FA oxidation inhibits PDH.^124^ However, activation of PDH by the mitochondrial Ca^2+^ overload occurring at reperfusion may overcome this inhibition and result in higher glucose oxidation than in normoxic conditions.^125^ Thus, glucose oxidation is reduced during early reperfusion following moderate ischaemia, but may increase following more severe or longer ischaemia.^114^, ^126^

Several ex vivo (ie in isolated perfused hearts) and in vivo studies have shown that stimulation of glucose oxidation during reperfusion is associated with a better recovery of myocardial function and a lesser reperfusion injury. In the ex vivo setting, interventions at reperfusion that increase glucose oxidation and improve the recovery of myocardial function may directly target the PDH flux, by activating PDH activity with dichloroacetate,^127^, ^128^, ^129^, ^130^ or by the law of mass action, provisioning extra pyruvate^129^ or increasing glucose uptake by administration of high glucose/high insulin at reperfusion^131^ or through overexpression of the GLUT1 transporter.^132^ Indirectly, inhibition of FA oxidation^133^, ^134^, ^135^ or uptake^136^, ^137^ may prevent inhibition of glucose oxidation by reversing the Randle mechanism. Importantly, IPC, the most robust cardioprotective intervention, was also shown to increase glucose oxidation during reperfusion,^138^, ^139^ although other studies found no changes.^140^

A major limitation of ex vivo experiments is that they do not allow assessment of long‐term post‐ischaemic myocardial salvage. Indeed, the improved recovery of function observed during the short reperfusion period (usually one hour) might reflect only speeding up of the mechanical recovery, associated with the improved re‐energization observed.^132^, ^133^, ^136^ Nevertheless, several ex vivo studies observed a reduction of myocardial necrosis biomarkers associated with increased glucose oxidation in response to glucose addition^141^ or FA uptake blockade.^136^

Fewer in vivo studies have addressed this question; yet, they have consistently reported a reduction in infarct size associated with stimulation of glucose oxidation by various mechanisms. Thus, one to several days after reperfusion following 30 minutes of coronary occlusion, the infarct size was reduced in response to acute administration prior to reperfusion of dichloroacetate,^142^ phosphonate compounds^143^ or reconstituted high‐density lipoproteins (HDL),^144^ which all stimulated glucose oxidation. Although administration of GLP‐1 analogue albiglutide^145^ or rosiglitazone days before the ischaemic event in diabetic rats^146^ also increased post‐ischaemic myocardial glucose oxidation and reduced infarct size, such approach is less relevant for the emergency room situation where patients cannot be treated beforehand.

Thus, most studies concur that stimulation of glucose oxidation during reperfusion improves the recovery of function and reduces infarct size. Conversely, in situations with reduced glucose oxidation, such as in pathologically hypertrophied hearts,^147^ a lesser recovery of function and/or larger infarct size is observed. There are, however, a few discordant observations, with studies indicating that stimulating PDH activity or reversing the Randle cycle may not be sufficient to limit post‐ischaemic injury.^131^, ^148^ Also, administration of Ruthenium Red, an inhibitor of mitochondrial Ca^2+^ uptake, after severe IR reduced PDH activation but improved the recovery of function and reduced creatine kinase release.^125^ Possibly, following severe ischaemia inducing massive post‐ischaemic Ca^2+^, the nefarious effects of mitochondrial Ca^2+^ overload overcome the positive effects of stimulating glucose oxidation.

Mainly two hypotheses are invoked to explain the positive impact of stimulating glucose oxidation on post‐ischaemic recovery of function and myocardial salvage: 1. efficiency of oxygen use and 2. reduction of proton overload. 1. Depending on the activity of the MAS, the complete oxidation of one glucose yields 31‐33 ATP, with a P/O ratio (ie moles of ATP produced divided by the moles of oxygen atoms used) of 2.6‐2.8.^149^ For complete oxidation of palmitate or oleate, the P/O ratio is only 2.45‐2.47. This 5% to 15% better oxygen efficiency of glucose oxidation over FA oxidation may not seem impressive, but may become critical when oxygen supply is limited or when mitochondrial function is deteriorated. 2. At least in isolated perfused hearts, the rate of glycolytic flux exceeds the rate of glucose oxidation, by up to one order of magnitude in the presence of FA.^128^, ^130^ Although complete glucose metabolism, that is glycolysis + oxidation, is proton neutral, glycolysis not followed by pyruvate oxidation generates two protons.^150^ During reperfusion, the glycolysis/oxidation uncoupling worsens, aggravating the proton overload, which drives the Na^+^ and Ca^2+^ overload leading to cardiomyocyte necrosis and/or opening of the mPTP.^151^ Stimulation of glucose oxidation without a concomitant stimulation of glycolysis, such as achieved with dichloroacetate, for example, reduces uncoupling and the generation of protons, thereby possibly limiting reperfusion injury. This interpretation puts uncoupled glycolysis as a driver of myocardial reperfusion injury, seemingly in contradiction with what was said in the previous section. Thus, stimulation of glycolysis could perhaps be a double‐edged sword, but the self‐harming edge can be blunted by concomitant stimulation of glucose oxidation.

In conclusion of this section, glucose oxidation appears to be a promising cardioprotection target that seems to have been overlooked by clinical studies. A search of the ClinicalTrials.gov database with the keywords ‘myocardial infarction’ or ‘reperfusion injury’ and ‘glucose oxidation’ failed to retrieve a single study. Acute stimulation of glucose oxidation at reperfusion with well‐tolerated agents such as dichloroacetate or reconstituted HDL would be worth trying in a clinical context.

#### 3.5.3 Fatty acid metabolism

The energy metabolism in heart heavily relies on fat oxidation. Transmembrane proteins such as CD36 and fatty acid transport proteins (FATPs) are involved in transport of nonesterified (free) long‐chain FA from circulation to cardiac tissues (Figure 1). Following cellular uptake, FA can either be stored in the form of triglycerides or undergo metabolism in mitochondria. Long‐chain (LC) FA metabolism proceeds through multiple steps ensuring transfer of LC acyl groups into mitochondria. The first step in this process is synthesis of acetyl‐CoA in the outer mitochondrial membrane.^152^ In the next step, carnitine palmitoyltransferase 1 (CPT‐1) catalyses synthesis of LC acylcarnitine which is necessary for transportation of fatty acid intermediates through the inner mitochondrial membrane (Figure 1).^153^ LC acylcarnitine synthesis rate is the FA metabolism rate‐limiting step which is regulated by concentrations of malonyl‐CoA, an endogenous inhibitor of CPT‐1.^154^ Malonyl‐CoA is synthesized by acetyl‐CoA carboxylase (ACC) from acetyl‐CoA using biotin and ATP as cofactors. ACC activity is regulated by its phosphorylation (inactivation) and dephosphorylation (activation). Accordingly, stimulation of fatty acid metabolism by AMPK is achieved by phosphorylation of ACC, yielding inhibition of malonyl‐CoA synthesis to ensure rapid LC acylcarnitine synthesis by CPT‐1.^155^ In contrast, activation of insulin signalling prevents ACC phosphorylation, stimulates malonyl‐CoA synthesis and results in CPT‐1 inhibition.^156^ Another enzyme, malonyl‐CoA decarboxylase (MCD), catalyses the reverse reaction and converts malonyl‐CoA into acetyl‐CoA. MCD activity is inhibited by SIRT4‐mediated deacetylation of the enzyme ensuring high malonyl‐CoA concentrations and facilitated LC acylcarnitine synthesis.^157^ LC acylcarnitines are further transferred from the intermembrane space into mitochondrial matrix and are converted to acetyl‐CoA by CPT‐2 to enter β‐oxidation in mitochondria.

The shift towards LC acylcarnitine accumulation in the mitochondria is a result of unbalanced AC synthesis and mitochondrial oxidation rates. As a consequence, CPT‐1 generates LC acylcarnitines at amounts which mitochondria cannot fully metabolize. During cardiac ischaemia, mitochondrial malfunction combined with energy deficiency‐driven activation of CPT‐1 results in even higher content of LC acylcarnitines. At high levels, LC acylcarnitines inhibit oxidative phosphorylation (OXPHOS), which in turn induces mitochondrial membrane hyperpolarization and stimulates the production of reactive oxygen species (ROS) in cardiac mitochondria (Figure 1).^158^, ^159^ Therefore, FA metabolism regulation approaches aiming at cardioprotection in IR settings should be carefully evaluated for effects on overall energy homeostasis and possible cardiotoxicity because of harmful fatty acid intermediate accumulation (ie ‘acyl‐carbon stress’). Some knockout mouse models altering transporters or enzymes involved in FA metabolism have resulted in disturbances in cardiac function.^160^ This highlights the importance of FA as essential substrates for the heart energy production to maintain normal cardiac function under chronic conditions. Nevertheless, the use of small molecular inhibitors of enzymes in fatty acid transport and metabolism pathways has demonstrated that inhibition of FA oxidation in general reduces damage induced by IR^160^, ^161^, ^162^, ^163^, ^164^, ^165^, ^166^, ^167^ (Table 1). It should be noted, however, that effects of FA on cardiac IRI are critically dependent on FA concentrations, with detrimental effects commonly only observed at rather high levels (>0.8 mmol/L) of FA.^168^ Although older literature has reported much higher FA plasma levels in cardiac patients, these high levels were likely the result of ongoing lipolysis in the test tube due to the use of heparin in these patients; preventing test tube lipolysis shows that these high FA levels are usually not observed during human in vivo IR episodes.^169^ Finally, FA may also contribute to cardiac IRI due to its dislodging effects on hexokinase II**‐**mitochondrial binding in the heart.^170^

**Table 1:** FAO inhibitors for IRI

Compound	Mechanism/target	Activity in MI models	Model	Ref
Sulfo‐N‐succinimidyl oleate (SSO)	Inhibition of sarcolemmal FAT/CD36	Prevented cardiac dysfunction after ischaemia	Isolated diabetic and control male Wistar rat hearts	[21]
CBM‐301940 CBM‐300864	Inhibition of malonyl‐CoA decarboxylase	Improved cardiac function during and after ischaemia	Isolated rat hearts Pigs in vivo	[22, 23]
Methyl‐GBB	Decreased accumulation of long‐chain acylcarnitines	Decreased MI size, improved survival	Ligation of LAD, rats Isolated perfused rat hearts	[24]
Trimetazidine	Long‐chain 3‐ketoacyl‐CoA thiolase inhibitor			
	AMPK and ERK signalling pathways	Reduced MI size and oxidative stress	In vivo regional ischaemia and reperfusion, mice	[25]
Carvedilol	Adrenergic receptor blocker; modulator of cardiac AMPK signalling pathway	MI size reduction, improved cardiac functions	Ligation of LAD, mice	[26]

Abbreviations: LAD, left anterior descending coronary artery; MI, myocardial infarction.

#### 3.5.4 Ketones

Beta‐hydroxybutyrate (BHB) and acetoacetate (AcAc) represent the two main ketone bodies.^171^ BHB, which is produced by degradation of FA in liver and then transported to extrahepatic tissues (including the heart), is one of the important metabolic substrates for energy production during fasting.^172^ BHB is not only a metabolic intermediate, but also possesses a variety of signalling functions.^173^ It has been shown to inhibit Class I histone deacetylases (HDACs), increasing therefore histone acetylation, and thereby induces the expression of genes that restrain oxidative stress.^174^ BHB also suppresses sympathetic nervous system activity and reduces total energy expenditure and heart rate by inhibiting short‐chain fatty acid signalling through G protein‐coupled receptor 41 (GPR41).^175^ The myocardium is the highest ketone body consumer per unit mass and under physiological conditions oxidizes ketone bodies in proportion to their delivery, at the expense of fatty acid and glucose oxidation.^176^, ^177^ Ketone bodies have an intermediate energetic efficiency, yielding more ATP per molecule of oxygen used (P/O ratio) in comparison with fatty acid oxidation, but less when compared to glucose.^178^, ^179^ Additionally, the oxidation of ketone bodies also yields potentially higher energy than fatty acid oxidation, keeping ubiquinone oxidized, which raises redox span in the electron transport chain (ETC) and makes more energy available to synthesize ATP. A recent ex vivo cardiac study employing direct measurements of cardiac efficiency showed no effects of ketones on cardiac efficiency.^9^ However, increasing the supply of ketones to the heart did increase total ketone body oxidation without a decrease in any other metabolic pathway.^9^ Therefore, ketone bodies are considered an alternative and efficient energy source in myocardium, especially in failing hearts.^180^ Increased circulating ketone bodies have been previously reported in patients with congestive heart failure.^181^

In addition to the beneficial effects of ketone bodies in heart failure, they have been shown to have beneficial effects in IRI by reducing myocardial infarct size, either by increased levels during fasting or when they were administered exogenously. Because of concentration‐dependent dynamics, increases in ketone bodies during fasting can elevate the rate of BHB utilization.^182^ It has been reported that fasting increased the myocardial BHB/AcAc ratio reflecting altered mitochondrial redox state and fasting of rats for only 24 hours improved the post‐ischaemic recovery of contractile function and reduced the lactate dehydrogenase release in isolated hearts subjected to global IR.^183^ In another study, short‐term fasting increased the concentration of BHB and BHB/AcAc ratio compared to controls, limited the infarct size and reduced the total number of premature ventricular complexes and the duration of ventricular tachycardia occurring at early reperfusion.^184^ However, low concentration of endogenous ketone bodies failed to preserve the myocardial ATP levels whereas exogenous supplementation (to 40 times the original concentration) prevented the loss of ATP by ischaemic injury.^185^

Administration of exogenous BHB 60 minutes before the start of ischaemia reduced in vivo myocardial infarct size and apoptosis in rats subjected to IR.^186^ It was recently demonstrated that starting in vivo BHB treatment at reperfusion and continuing administration for the next 24 hours of reperfusion using minipumps reduced infarct size, attenuated apoptosis in myocardium and preserved cardiac function of IR in mice. The above‐mentioned beneficial effects were attributed to reduced mitochondrial formation of ROS, enhanced ATP production, attenuated mitochondrial swelling and partly restored mitochondrial membrane potential in myocardium.^187^

In summary, in the experimental IRI context, ketone bodies may confer cardioprotective effects possibly due to altered mitochondrial redox state resulting from increased ketogenesis, up‐regulation of crucial OXPHOS mediators and reduction of oxidative stress.

#### 3.5.5 Succinate and ROS

A new mechanism is emerging whereby the production of mitochondrial ROS is considered a highly orchestrated, metabolite‐driven process early in IRI. The citric acid cycle metabolite, succinate, is extensively accumulated during ischaemia and is rapidly oxidized upon reperfusion.^79^, ^188^, ^189^ In v*ivo*, succinate likely accumulates via the reduction of fumarate by succinate dehydrogenase (SDH or complex II) reversal. A lack of the terminal electron acceptor, oxygen, maintains a reduced CoQ pool, and additionally, the pH of ischaemic tissue is lowered.^51^, ^79^, ^190^ Upon reperfusion, succinate is rapidly oxidized to fumarate, and together with ETC activity restarting, the reduced CoQ pool provides electrons for the ETC complex to proton pump, establishing a large proton motive force (Δp). The large Δp and highly reduced CoQ pool, together with depleted adenine nucleotides, drive reverse electron transport (RET) through mitochondrial complex I, resulting in the production of superoxide at the flavin mononucleotide (FMN) site (Figure 1).^191^, ^192^ The mitochondrial ROS produced, together with impaired calcium handling, activate downstream pathways, resulting in mitochondrial permeability pore formation and, ultimately, cell death.

Not all of the succinate that has accumulated at ischaemia is oxidized by SDH, as it has been suggested that a proportion is released from the cell.^75^ Succinate was significantly elevated in the blood of patients with an acute ST**‐**elevation myocardial infarct^193^ suggesting its release into the bloodstream upon reperfusion. This opens the intriguing possibility that changes in mitochondrial metabolites during IRI could be involved in paracrine signalling, complementary to the signalling role succinate plays in the immune system.^194^

Accumulation of succinate, with its derivative succinyl‐CoA, also leads to protein succinylation.^195^ The Sirt5, which has limited deacylase activity, also catalyses the removal of succinyl groups from proteins. In line, it has been shown that the increase in IRI in Sirt5‐deficient mouse heart can be reversed by preventing succinate accumulation.^196^

Blocking succinate metabolism during ischaemia or reperfusion with malonate, a competitive SDH inhibitor, has been found to be protective in multiple pre‐clinical models of cardiac IRI in mouse, rat or pig.^51^, ^188^ Therefore, targeting succinate metabolism through SDH inhibition is twofold, either by preventing the succinate rise during ischaemia or by reducing the oxidation upon reperfusion, highlighting a potential therapeutic target.^197^

At present, the metabolic source for succinate in vivo appears to be fumarate. However, a key question remains as to the mechanism of fumarate production during ischaemia. Firstly, adenosine monophosphate (AMP) build‐up during ischaemia can be broken down to fumarate via the purine nucleotide cycle (PNC). Secondly, the conversion of aspartate to oxaloacetate and then reduction to malate in the MAS could provide the fumarate required.^51^, ^112^ Alternatively, a recent mechanism has suggested that canonical TCA cycle activity may result in succinate accumulation, by aminotransferase anaplerosis, as opposed to SDH reversal.^75^ Differences in the models used to investigate metabolic changes may be resulting in discrepancy as to which direction of the TCA cycle contributes most to ischaemic succinate accumulation. Further work is required to elucidate accumulation pathways. However, the mechanistic insights produced by further investigating succinate in IRI will provide key targets for the design of cardioprotective drugs, thus providing many lucrative avenues for future therapies in many pathologies.

Importantly, the role of succinate as a proximal source of electrons for ROS generation during reperfusion is undisputed. As such, the acute delivery of complex II inhibitors at the onset of reperfusion, regardless of succinate accumulation, appears to be a potentially promising clinical approach for treatment of MI in the acute setting (eg during PCI, percutaneous coronary intervention).

#### 3.5.6 F_O_F_1_‐ATPase **during ischaemia**

Mitochondrial ATP synthase or FoF_1_‐ATP/synthase transforms the electrical power generated during respiration (ΔΨm) into ATP‐containing chemical energy, following the chemiosmotic principle that governs the life of all organisms. It contains an H^+^ channel domain (Fo) embedded within the inner mitochondrial membrane and a catalytic domain (F_1_) protruding towards the mitochondrial matrix, interconnected by central and peripheral stalks.^198^ High‐resolution cryoelectron microscopy of native mitochondrial membranes has revealed that FoF_1_‐ATP/synthase self‐associates into long rows of dimers that shape the cristae of mitochondria of all eukaryotic cells into elongated tubular cristae.^199^ Adequate shaping of mitochondrial cristae determines the respiratory fitness.^20^ The FoF_1_‐ATP/synthase is extremely efficient in generating ATP (30 kg/day in a healthy heart), but when cardiomyocytes are challenged by an anoxic episode, the catalytic subunit may paradoxically reverse into an energy‐dissipating machine, favouring H^+^ extrusion at the expense of ATP hydrolysis.^21^

Under physiological conditions, a fraction of FoF1‐ATP/synthase remains blocked by the inhibitory factor 1 (IF1), a 12 kDa protein that translocates exclusively to the mitochondria. Remarkably, these tissues with high‐energy demand, like the heart, have the highest content of IF1, probably because in these tissues a relevant fraction of the FoF1‐ATP/synthase only becomes activated upon demand, acting as a reservoir for ATP synthesis. Expression of IF1 varies between species, and as a general rule, it is higher in animals with a high rate of heart contraction, like mice, and lower in species with low rate of heart contraction, like humans.^22^ Under ischaemic conditions, reversion of FoF1‐ATP/synthase into a hydrolase precipitates energy exhaustion and rigour contracture in cells already jeopardized by the lack of oxygen.^23^ Therefore, species‐dependent expression levels of IF1 can affect the susceptibility of cardiomyocytes to ischaemic damage.^24^ Moreover, IF1 has been involved in metabolic reprogramming: By impeding ATP synthesis, it can inhibit OXPHOS and drive the cell towards a more glycolytic metabolism.

Cardiomyocytes from aged mice exhibit a partial failure of FoF1‐ATP/synthase to revert its catalytic mode of operation^34^; this alteration delays the development of ischaemic‐rigour contracture secondary to ATP exhaustion but accelerates (ΔΨm) decline during ischaemia and impairs (ΔΨm) recovery upon reperfusion in the aged cardiomyocytes, a response that is paralleled by more pronounced mPTP opening, hypercontracture and cardiomyocyte death.^34^ Indeed, recent evidence suggests that FoF1‐ATP/synthase could be the true molecular entity of the mPTP (Figure 1), the opening of which has been consistently associated with the extension of myocardial necrosis during ischaemia‐reperfusion injury (IRI) in different experimental models.^25^ A structural alteration in the C‐ring within FoF1‐ATP/synthase^26^ or in the molecule dimerization^27^ has been proposed to act as ‘death channel’, with the latter receiving a greater consensus and more experimental support.^28^ An intermediate model includes the dissociation of FoF1‐ATP/synthase dimers followed by the rearrangement of the C‐ring.^29^ The concept that FoF1‐ATP/synthase is the anatomical support for H^+^ dissipation and cell death integrates other well‐accepted mPTP regulators,^210^ including CypD, which has been recently proposed to reduce mito‐ATP synthase supramolecular assembly, thereby increasing mPTP opening probability.^211^ Mass spectrometry methods have detected several types of post‐translational modifications in different subunits of FoF1‐ATP/synthase,^212^ some of them, that is, phosphorylation of β‐subunit, have been detected in response to adenosine‐induced conditioning strategy.^213^ Nevertheless, to date, the impact of FoF1‐ATP/synthase post‐translation modifications on the susceptibility of cardiomyocytes to undergo mPTP and death upon IRI has not been established. Because mPTP is increasingly recognized as a prominent therapeutic target in the context of myocardial IRI, the elucidation of the role of FoF1‐ATP/synthase as a modulator of cardiomyocyte death and survival may help to identify new pharmacological strategies for cardioprotection.

### 3.6 Energy transfer pathways

Mitochondria are central players of cellular energy metabolism, especially in striated oxidative muscles and heart. This is much more complicated than only production of ATP via OXPHOS, located on mitochondrial inner membrane (MIM). Mitochondria are also source of ROS, proapoptotic factors; they synthesize different metabolites, regulate cellular redox potential and play an important role in ion homeostasis regulation and thermogenesis. Severe myocardial infarction leads to heart failure due to a marked loss of functional activities of cardiomyocytes, where reorganization of energy transport pathways is an important component.

Studies in the last decades have led to an understanding that in cardiomyocytes, the cellular energy metabolism is a precisely organized system where mitochondria and ATPases are linked to each other by specialized energy transfer pathways formed by isoenzymes of creatine kinase (CK) and adenylate kinase (AK) and glycolytic enzymes like hexokinase (HK). In addition to the regulation of cellular respiration by calcium homeostasis, CK and AK energy transfer pathways ensure precise feedback signalling between contraction workload and oxygen consumption in mitochondria. Each sarcomere has its own corresponding mitochondrion, which together with phosphotransfer system and feedback metabolic signalling creates the intracellular energy unit (ICEU).^214^, ^215^, ^216^

An important characteristic of the heart is its metabolic stability, as reflected by the apparent invariability of intracellular concentration of ATP and phosphocreatine (PCr) in spite of the variable workloads, corresponding rates of ATP oxidative synthesis and myofibrillar hydrolysis.^217^ Under conditions of total ischaemia, the PCr concentration falls rapidly and heart contraction ceases, but ATP concentration stays almost stable decreasing only by 10% at the end of the first minute of ischaemia.^217^ Colocalization of BetaII‐tubulin and VDAC in heart muscle is functionally related to the ability of creatine to stimulate OXPHOS due to functional coupling between mitochondrial creatine kinase (MtCK) and adenine nucleotide translocase (ANT).^218^ Key events observed after acute ischaemia‐reperfusion (IR) and chronic ischaemia are the decrease (or loss) in the stimulatory effect of creatine and decrease in diffusion restrictions for ATP and ADP at the level of the mitochondrial outer membrane (MOM), which is mediated by BetaII‐tubulin^219^, ^220^ The disruption of mitochondrial interactions with cytoskeleton will result in decreased intracellular compartmentalized energy transfer and the loss of probability of interaction between mitochondria and BetaII‐tubulin. In adult cardiomyocytes, octameric mitochondrial creatine kinase (MtCK) binds electrostatically to the negatively charged cardiolipins of the mitochondrial inner membrane sharing the same cardiolipin patches with ANT.^221^ It is possible that the unaltered octameric isoenzyme of MtCK ensures the stability of its molecular interaction with ANT after IR.^220^ Alternatively, the IR‐induced alteration of mitochondrial increased MOM permeability might influence kinetic properties of the MtCK.^220^

The predominant HK isoform in adult heart, HK2, dynamically shuttles between the mitochondria and cytoplasm, resulting in increased glycolysis when bound to mitochondria.^222^ Besides that, mitochondrially bound HK2 is an important player in the field of voltage‐dependent anion channel (VDAC) interactions with regulatory proteins and its functional coupling with OXPHOS. It was recently shown that IR disrupts interactions between VDAC, ANT and HK2 through nitration of tyrosine residues in VDAC and ANT, contributing to mitochondrial and cellular dysfunction following IR.^223^ Contact sites between MIM and MOM have a substantial role in transporting ATP, generated within the mitochondria, to the cytosol as PCr. Binding between CK and HK2 may stabilize these contact sites, and loss of HK2 during ischaemia leads to contact site breakage and decreased rates of extramitochondrial PCr synthesis.^36^, ^224^, ^225^ The interplay between energy transfer pathways and different binding sites for tubulin and hexokinase to VDAC may be one of the targets of IPC.

The connections of mechanisms of energy transfer pathways and cardioprotection are not clear yet; one component participating in this system is the AK system together with K_ATP_ channel. The AK‐catalysed phosphotransfer system would promote K_ATP_ channel opening primarily by accelerating conversion of ATP to ADP, whereas CK systems would predominantly facilitate conversion of ADP to ATP and K_ATP_ channel closure.^226^

The alterations in MOM permeability for adenine nucleotides seem to be an important feature of cardiac ischaemic and IR injuries, as it regulates the energy transfer pathways. IPC induced redistribution of high‐energy phosphoryl transfer and increased phosphotransfer reactions (creatine kinase, glycolysis), leading to improved intracellular metabolic communication and preservation of cellular ATP synthesis and ATP consumption following IR.^227^ These phosphoryl fluxes correlated tightly with post‐ischaemic functional recovery^227^ and pre‐conditioning‐induced energetic remodelling, improving contractile performance following IR. The study of intracellular phosphotransfer reactions is still not fully unravelled and will need more sophisticated studies, before it can be used in the development of an injury‐tolerant state in cardiomyocytes.

## 4 CONCLUSION

We have outlined some of the important metabolic changes occurring during ischaemia and subsequent reperfusion. While many aspects happen in the entire cell, the mitochondria are a clear focus within many of these metabolic changes. Such changes include alterations in fatty acid and succinate metabolism, along with F_O_F_1_‐ATP/synthase activity, resulting in increased mitochondrial ROS production and subsequent opening of mPTP. Additionally, glycolysis, hexosamine biosynthesis, glucose oxidation ketone metabolism and the malate/aspartate shuttle are all processes which directly affect metabolite levels and mitochondrial pathways. All of which have shown to elicit cardioprotective effects when altered.

While many of the outlines metabolic processes are ideal drug targets for ischaemia/reperfusion injury, further studies are necessary to fully understand underlying mechanisms and establish potential therapies. In this context, it is vital to test possible pharmacologic interventions at a clinically relevant time‐point at the end of ischaemia as well as in pre‐diseased and aged models.^228^ Some of the outlined mechanisms have already been shown to be relevant in man^193^ and targeted successfully in many species, including large animals.^189^, ^197^ Others are still awaiting a relevant drug target suitable for translation in patient with an acute MI. Recent methodological advances in detecting metabolic changes within the heart will make these efforts easier to achieve. Furthermore, the observed metabolic changes are not limited to cardiac I/R injury, but could play important roles in many physiological and pathophysiological situations, such as exercise, inflammation and cancer.

## DISCLOSURES

None.

## AUTHOR CONTRIBUTIONS

Each author wrote part of the review and edited the entire manuscript.
