# CORRIGENDUM

**DOI:** 10.1111/jcmm.16095

**Published:** 2021-01-17

In Zuurbier et al,^1^ the published article contains errors in Table 1. The correct table is shown below. The authors confirm all results, conclusions of this article remain unchanged.

**TABLE 1:** FAO inhibitors for IRI

Compound	Mechanism/target	Activity in models of MI	Model	Ref
sulfo‐N‐succinimidyl oleate (SSO)	Inhibition of sarcolemmal FAT/CD36	Prevented cardiac dysfunction after ischaemia	Isolated diabetic and control male Wistar rat harts	[161]
CBM‐301940 CBM‐300864	Inhibition of malonyl CoA decarboxylase	Improved cardiac function during and after ischaemia	Isolated rat hearts Pigs in vivo	[162, 163]
Methyl‐GBB	decreased accumulation of long‐chain acylcarnitines	Decreased MI size, improved survival	ligation of LAD, rats Isolated perfused rat hearts	[164]
Trimetazidine	long‐chain 3‐ketoacyl‐CoA thiolase inhibitor AMPK and ERK signalling pathways	Reduced MI size and oxidative stress	in vivo regional ischaemia and reperfusion, mice	[165]
carvedilol	adrenergic receptor blocker; modulator of cardiac AMPK signalling pathway	MI size reduction, improved cardiac functions	ligation of LAD, mice	[166]

Abbreviations: LAD, left anterior descending coronary artery; MI, myocardial infarction.
